# Proteomic Approaches in Understanding Action Mechanisms of Metal-Based Anticancer Drugs

**DOI:** 10.1155/2008/716329

**Published:** 2008-07-22

**Authors:** Ying Wang, Jen-Fu Chiu

**Affiliations:** Department of Anatomy, The University of Hong Kong, PokFuLam Road, Hong Kong SAR, China

## Abstract

Medicinal inorganic chemistry has been stimulating largely by the success of the anticancer drug, cisplatin. Various metal complexes are currently used as therapeutic agents (e.g., Pt, Au, and Ru) in the treatment of malignant diseases, including several types of cancers. Understanding the mechanism of action of these metal-based drugs is for the design of more effective drugs. Proteomic approaches combined with other biochemical methods can provide comprehensive understanding of responses that are involved in metal-based anticancer drugs-induced cell death, including insights into cytotoxic effects of metal-based anticancer drugs, correlation of protein alterations to drug targets, and prediction of drug resistance and toxicity. This information, when coupled with clinical data, can provide rational basses for the future design and modification of present used metal-based anticancer drugs.

## 1. METAL-BASED ANTICANCER DRUGS

Medicinal applications of metal complexes as therapeutic drugs have a more than 5000-year history [1]. Since the discovery of the anticancer activity of cisplatin by Shimizu and Rosenberg 35 years ago, there has been a rapid expansion in research to find new, more effective metal-based anticancer drugs [2]. The major classes of metal-based anticancer drugs include platinum (II), gold (I) and gold (III), metalloporphyrin, ruthenium (II) and ruthenium (III), bismuth (III), rhenium (I), and copper (II) compounds.

### 1.1. Platinum(II) anticancer drugs

Cisplatin represents one of the most potent drugs available in the cancer chemotherapy for several solid tumors, such as testicular, ovarian, bladder, and neck cancers [3]. It is generally believed that cisplatin exhibited its anticancer effects through preferentially binding to quinine N-7 of DNA, and then cause DNA damage specifically in cancer cells, subsequently leading to cell death [4]. After successes achieved with platinum complexes, there is a tremendous increase in the search for platinum complexes with different ligands that might produce more specific anticancer effects. Some of these platinum-based drugs have been approved by the Food and Drug Administration (FDA), including carboplatin for the treatment of ovarian cancer [5, 6], oxaliplatin for metastatic colorectal cancer [7, 8], satraplatin for hormone-refractory prostate cancer [9], and picoplatin for small-cell lung cancer [10].

Transplatinum compounds follow different patterns of cell killing in comparison to cisplatinum, thus giving a reason for optimism in their development as a new class of platinum-based anticancer drugs [11]. The initial report of anticancer properties of a dinuclear platinum complex in 1988 started a new paradigm in platinum-based chemotherapy. Several multinuclear platinum complexes have entered clinical trials in recent years, with varying results [12, 13]. The major limitations of cisplatin and other platinum anticancer drugs are related to drug resistance and their side effects, including nephrotoxicity, neurotoxicity, and emetogensis [14]. Resistance to cisplatin is multifactorial, most cases consist of mechanisms limiting the formation of DNA adducts or operating downstream of the cisplatin-DNA interaction to promote cell survival [15].

### 1.2. Gold (I) and gold (III) anticancer complexes

Gold (I) complexes had been used for the treatment of arthritis some decades ago, but most of them disappeared from the drug market because of intolerable side effects, such as gastrointestinal adverse reactions, nephrotoxicity, and haematological reactions. However, the design and testing of gold complexes, especially gold (III) complexes with anticancer activity begin to be intensively pursued in the past few years. The potential use of gold (III) complexes as anticancer drugs were based on three rationales [16–18]: (a) analogies between square planar complexes of both platinum (II) and gold (III) are d^8^ ions; (b) analogy to the immunomodulatory effects of gold (I) antiarthritic agents; and (c) complexation of gold (I) and gold (III) with known anticancer agents to form new compounds with enhanced activity. Buckley et al. first reported some organogold (III) complexes endowed with significant cytotoxic and anticancer properties [19]. During the past decades, various gold (III) complexes with sufficient stability in the physiological environment have been synthesized and evaluated for in vitro anticancer properties. Some of these gold (III) complexes turned out to exhibit relevant cytotoxic effects in vitro and were the subject of further biochemical and pharmacological investigations [20–33]. Our previous findings showed that gold (III) mesotetraarylporphyrin 1a was stable against demetallation in physiological conditions and exhibited higher cytotoxicity than cisplatin against a panel of human cancer cell lines [34–37]. The major limitation of gold (III) complexes is that few exhibit good stability under physiological conditions, due to the reduction of gold (III) to gold (I) [38]. However, low cisplatin cross resistance has been observed in gold complexes [39]. There is therefore considerable interest in the development of tumor-selective and stable gold anticancer drugs.

### 1.3. Metalloporphyrin drugs

Metalloporphyrin drugs are new class of antioxidant enzyme mimetics with novel structure: a metal in the center of porphyrin ligand. Metalloporphyrins (e.g., MnTBAP) have previously been used to inhibit age-related oxidative damage in myocardium of mice that are lacking mitochondrial enzyme manganese superoxide dismutase [40]. Afterwards, metalloporphyrin drugs began to be used as photodynamic therapy agents for certain solid tumors [41]. Photodynamic therapy is based on the concept that porphyrins are known to be rapidly and preferentially taken up by the tumor cells with higher intakes of lipoproteins [42, 43]. When such photosensitizers are irradiated with an appropriate wavelength of visible or near infrared (NIR) light, the excited molecules can transfer their energy to molecular oxygen in the surroundings, which is normally in its triplet ground state. This results in the formation of cytotoxic reactive oxygen species (ROSs), particularly singlet oxygen [44]. ROSs are responsible for oxidizing various cellular compartments including plasma, mitochondrial, lysosomal, and nuclear membranes, resulting in irreversible damage of tumor [34, 44]. Therefore, under appropriate conditions, photodynamic therapy offers the advantage of an effective and selective method of destroying diseased tissues without damaging adjacent healthy cells [42, 43].

Since the approval of Photofrin by FDA for chemotherapy [45], porphyrin derivatives with different metal in the center of the molecule have been widely used as photosensitizers for photodynamic therapy in the treatment of cancer, including chlorophyllin copper complex as superoxide dismutase mimics [46, 47], FeTBAP and MnTBAP [48, 49], ZnTBAP [50], motexafin gadolinium (MGd) [51]. Different modes of actions have been suggested for different kinds of metalloporphyrins. For example, MGd has been shown to inhibit heme oxygenase-1 (HO-1) activity that results in inactivation of the antiapoptotic properties of the products of HO-1 [51]. While FeTBAP and MnTBAP have been reported to be superoxide anion scavengers [48]. MGd is also an active inhibitor of cytochrome P450 enzymes, although with a lower potency than that exhibited for inhibition of HO-1.

### 1.4. Other metal-based anticancer drugs

In recent years, other approaches in the search for new, metal-based anticancer agents are to examine complexes that contain other transition metals. In the design of these new drugs, octahedral ruthenium (II) and ruthenium (III) complexes have shown antineoplastic properties on a number of experimental tumors. Tetraammine-, pentaammine-, heterocycle-, and dimethylsulfoxide-coordinated ruthenium complexes have been synthesized and shown high affinity for nitrogen donor ligands in vitro and as a result exhibit anticancer action in vivo [52–54]. Other transition metals have been used as anticancer drugs, including bismuth (III) labeled antibodies for systemic radioimmunotherapy [55, 56], rhenium (I) complexes as DNA-binding agents [57], (MTR)_2_Zn^2+^ complex that induces cancer cell death by binding to chromatin [58], and Cu^2+^ compound chlorophyllin initiated apoptosis in human colon cancer cells through caspase-8 and apoptosis-inducing factor (AIF) activation in a cytochrome *c*-independent manner [46].

## 2. PROTEOMICS

### 2.1. Introduction to proteomics

The proteome is defined as all expressed protein complement of a cell, organ or organism, and it includes all isoforms and posttranslational variants. Proteomic technology, first coined in 1995 [59], attempts to separate, identify, and characterize a global set of proteins in an effort to provide information about protein abundance, location, modification, and protein-protein interaction in the proteome of a given biological system [59, 60]. This postgenomic technology provides a direct measurement of the presence and relative abundance of proteins, and reveals the consequence of protein functioning in establishing the biological phenotype of organisms in different states. By studying interrelationships of protein expressions and modifications in health and disease or drug treatment, proteomics contributes important insights into determining the pathophysiological basis of disease [61], validating drug targets [62], and illustrating drug action [63], toxicity, and side effects [64].

### 2.2. Technological platforms

In the field of proteomics, several well-established methods persist as means to resolve and analyze complex mixtures of proteins derived from cells and tissues. Currently, the most commonly used proteomic platforms include two-dimensional gel electrophoresis (2DE) and protein chip arrays, isotope-coded affinity tages (ICATs), and immobilized metal affinity chromatography (IMAC) (Table 1). These technological platforms are most often incorporated with matrix-assisted laser desorption/ionization time of flight (MALDI-TOF) [65], surface enhanced laser desorption ionization time of flight (SELDI-TOF) [66], electrospray ionization (ESI) [67], and/or tandem mass spectrometry (MS/MS). In addition, inductively coupled plasma (ICP) mass spectrometry has also been applied in proteomic-based research of drug discovery [68].

## 3. POTENTIAL APPLICATIONS OF PROTEOMICS IN ILLUSTRATING METAL-BASED DRUG DEVELOPMENT AND DISCOVERY

Ever since the initial discovery of the anticancer activity of cisplatin, major efforts have been devoted to elucidate the biochemical mechanisms of anticancer activities of metal-based drugs to facilitate rational design of novel metal-based drugs with better pharmacological profiles. A comprehensive understanding of the molecular action mechanisms, which are triggered by metal-based drugs to kill cancer cells can lead to the design of more effective anticancer drugs, as well as to provide new therapeutic strategies based on the molecular activity of metal-based drug activity.

### 3.1. Target discovery and validation

Target discovery, which involves the identification and early validation of disease-associated targets, is an essential first step in the drug discovery pipeline [81]. Indeed, the drive to determine protein function has been stimulated, both in industry and academia, by the human genome and proteome projects in progress. Proteomics, the study of cellular protein expression, is an evolving technology platform that has the potential to identify novel proteins involved in key biological processes in cells. These proteins may serve as potential drug targets. Proteomics thus holds great promise as a powerful technique for drug target discovery. It must be pointed out, however, that numerous drug-targeted proteins are membrane-bound proteins, for example, receptors and ion channels. These proteins may not be amenable for study by proteomics due to their poor solubility and low abundance, and thus they are disproportionally represented in proteome profiles [82]. Up to date, only a fraction of putative drug targets has been identified by proteomic approaches, including the volume-sensitive organic osmolyte/anion channel as key elements of tumor development, migration, and invasiveness [83], and integrin alpha-4 as a molecular target of oxidative stress [84].

Studying protein expression profiling of drug-treatment leads to the identification of a number of drug-specific targets both in vivo and in vitro. Using HPLC-MALDI-TOF MS, Hasinoff et al. have identified topoisomerase II*α* contained at least five free cysteins (170, 216, 300, 392, and 405) and two disulfide-bonded cyteine pairs (427-455 and 997-1008) [85]. Cisplatin was found to antagonize the formation of a fluorescence adduct between topoisomeraser II*α* and the sulfhydryl-reactive maleimide reagent 10-(2,5-dihydro-2,5-dioxo-1H-pyrrol-1-yl)-9-methoxy- 3-oxo-3H-naphtho[2,1-b]pyran-2-carboxylic acid methyl ester (ThioGlo-1). Based on these results, the authors suggested that topoisomeraser II*α* cysteines may be possible sites responsible for the inhibition of the catalytic activity of topoisomeraser II*α* observed in the presence of cisplatin, and topoisomeraser II*α* cysteins and DNA as targets responsible for cisplatin-induced inhibition of topoisomeraser II*α* [85]. Besides, mitochondrial proteins, especially ATP synthase-beta subunit, have been reported to be key proteins that serve as primary target of manganese porphyrin [MnTnHex-2-PyP(5+)] treatment during renal ischemia/reperfusion injury by proteomic study [86].

ICP MS coupled with capillary electrophoresis (CE) has been used in the identification, characterization, and determination of different chemical species of an element in complex biological systems, that is, the impetus of biochemical speciation analysis [87, 88]. Polec-Pawlak et al. have used this approach to study the platinum group metallodrug-protein binding aiming to characterize the interactions between cancer-inhibiting metal complexes and serum transport proteins [89]. Such binding does not only regulate the uptake and accumulation of the drug in tumor tissue but also determines its overall distribution and exertion and differences in efficacy, activity, and toxicity [90, 91]. Their study provides clear evidence that a ruthenium (III) complex [trans-tetrachlorobis(1H-indazole)ruthenate (III)] (KP1019) preferentially binds toward albumin whose adduct is a dominating protein-bound species of ruthenium [89].

### 3.2. Validation of drug toxicity and resistance

Insights into toxic responses are an asset for the interpretation of adverse drug effects and contribution to accurate risk assessment for humans. Proteomics in combination with combinatorial chemistry and high-throughput screening can help to bring forward validating toxicity and resistance of an unprecedented number of potential lead compounds [92]. Benefits can be expected in optimized clinical trials based on the availability of biologically relevant markers of drug efficacy and safety. Proteomics has demonstrated proof-of-concept in toxicology as shown by a number of successful applications in mechanistic toxicology and lead selection. Proteomic studies of liver toxicity have been carried out with thioacetamide [93] and ethanol [64]. Other researches have studied nephrotoxicity of cyclosporine A by proteomic approaches [94, 95] and reported a profound downregulation of the calcium binding protein calbindin D28 responsible for cyclosporine A-induced kidney toxicity. However, this technology-driven acceleration in drug discovery moves the bottlenecks in drug development to the downstream, which is the improvement in the selection of patient populations for clinical trials.

Proteomic approaches have been used to identify the underlying mechanisms for cisplatin resistance [96]. In this study, the authors used cervix squamous cell carcinoma cell line A431 and its cisplatin-resistant subline, A431/Pt as model system. The identified differentially expressed proteins can be classified into several groups, including molecular chaperones (e.g., heat-shock protein HSP60, HSP90, and HSC71), Ca^2+^-binding proteins (e.g., calmodulin and calumenin), proteins involved in drug detoxification (e.g., peroxiredoxin 2 and 6, and glutathione-S-transferase), antiapoptotic proteins (e.g., 14-3-3 switched on in cisplatin-exposed cells) and ion channels (e.g., voltage-dependent anion channel 1, voltage-dependent anion-selective channel). Besides this, proteome profile of cisplatin sensitive ovarian cell line IGROV1 and its cisplatin-resistant counterpart IGROV1-R10 have been compared aiming to find any protein markers or to establish new therapeutic strategies [97, 98]. Increased expression of cytokeratin 8 and cytokeratin 18 was considered to play a role in acquired chemoresistance of IGROV1-R10 cancer cell line to cisplatin [97, 98]. Cytokeratin 8 and cytokeratin 18 have been implicated in resistance to TNF-*α*-induced apoptosis by binding the cytoplasmic domain of tumor necrosis factor receptor 1 [69, 99]. Moreover, human nasopharyngeal carcinoma cells deficient for cytokeratin 8 were more sensitive to cisplatin-induced apoptosis [100].

In addition, acquired and intrinsic cellular drug resistances are multifactorial processes, involving induction of drug detoxifying mechanisms, quantitative and qualitative modification of drug targets, cell cycle arrest, regulation of DNA replication or reparation mechanisms, modulation of apoptosis, and other mechanisms [101, 102]. Global examination of the glycoproteomes of the cisplatin-resistant ovarian cancer cell line IGROV-1/CP using shotgun glycopeptide capture approach coupled with MS has been used to study cisplatin resistance [103]. In this approach, glycopeptides derived from glycoproteins are enriched by selective capture onto a solid support using hydrazide chemistry followed by enzymatic release of the peptides and subsequent analysis by MS/MS. This method improves solubility of large membrane proteins and exposes all of the glycosylation sites to ensure equal accessibility to capture reagents. Stewart et al. also used isotope-coded affinity tags (ICATs) integrated with mRNA expression levels to study cisplatin resistance in ovarian cancer cells [104]. Their study identified three pathways in Panther database (http://www.pantherdb.org/) that were significantly (*p* < .05) upregulated in cisplatin-sensitive cells, including glycolysis, interleukin signaling pathway, and PI 3-kinase pathway [104].

### 3.3. Mapping drug action mechanisms

An understanding of protein function within the context of complex cellular networks is required to facilitate the discovery of novel drug targets and, subsequently, new therapies directed against them. Proteomics offers comprehensive monitoring of protein alterations at molecular level upon drug treatments. Being the basic biochemical mode of drug activities, drug action mechanism should be better understood to provide valuable insights into drug modification and new drug development [34, 105]. Successful examples in drug mechanism study using proteomics include the illustration of insulin-like growth factor-binding protein-6-induced sublethal hydrogen peroxide stress in human diploid fibroblasts cells [106]. By using ESI MS/MS, Kanski et al. have applied proteomic analysis of protein nitration in aging skeletal muscle and identified nitrotyrosine-containing sequences in vivo [107].

In our previous study, we have used 2DE-based proteomic technology to compare the protein profile of human nasopharyngeal carcinoma SUNE1 cell line treated with gold (III) porphyrin 1a, and a number of differentially expressed proteins were identified [35]. These proteins can be classified into several categories based on their major biological functions, including cellular structural proteins, stress-related and chaperone proteins, proteins involved in ROS, enzyme proteins and translation factors, proteins that mediate cell death and survival signaling, and proteins that participate in the internal degradation system [35]. Among these proteins, one of the significant increased proteins is voltage-dependent anion channel 1 (VDAC 1). VDAC 1 is a mitochondrial outer membrane channel protein, which functions as the pathway for the movement of various substances in and out of the mitochondria [108]. It is considered to be a component of the permeability transition pore oligoprotein complex that plays a role in the permeability transition [109, 110]. VDAC 1 also plays an essential role in Bax/Bak-induced apoptotic mitochondrial changes in the process of mammalian cell death [111–113]. In this process, the proapoptotic proteins Bax and Bak bind to VDAC 1, and enhance its permeability so that cytochrome c passes through the channel and releases to cytoplasm [111–113]. Our data on VDAC 1 upregulation [35] and Bax overexpression [37] suggest that gold (III) porphyrin 1a may induce cell death via the mitochondria-mediated apoptosis pathway. Further functional studies revealed that gold (III) porphyrin 1a caused depletion of mitochondrial transmembrane potential (ΔΨ_m_) soon after uptake with suppression of Bcl-2, and activation of caspase 9 and caspase 3 [34]. Taken together, these results suggested that mitochondria are the primary target of gold (III) porphyrin 1a.

Quantitative proteomic analysis on other metal-based anticancer drugs has also been pursuit. Schmidt et al. have used nano-LC coupled offline MALDI-TOF/TOF-MS to study cisplatin-induced apoptosis in Jurkat T cells [114]. Their results showed that this method is more accurate than the commonly used online LC-ESI-MS.

## 4. CONCLUSION AND FUTURE PROSPECTS

The potential value of proteomics in metal-based drug development, especially in mapping drug action mechanisms, has been demonstrated in many successful examples. Proteomic approaches have been recognized as promising techniques that can facilitate the systematic characterization of a drug targets' physiology, thereby helping to reduce the typically high attrition rates in discovery projects, and improving the overall efficiency of pharmaceutical research processes. However, at present stage, the bottleneck for taking full advantage of this new experimental technology is the rapidly growing volumes of automatically produced biological data, and technical challenges with regards to sampling, tumor heterogeneity, and lack of standardized methodologies. In addition, to complement the limitation of current proteomic technology, systematic biological and pharmaceutical studies should be integrated with proteomics to better serve the purpose of illustrating the action mechanism of drugs and thus contribute to the success in metal-based drug development.

## Figures and Tables

**Table 1:** Major technological platforms in proteomics.

*Technique*	*2DE* (Two-dimensional gel electrophoresis)	SELDI-TOF MS	*ICAT* (Isotope-coded affinity tags)	*IMAC* (Immobilized metal affinity chromatography)
Principles	2DE separates protein mixtures by their isoelectric points and molecular weights, proteins can be identified by MALDI-TOF MS through enzyme digestion	This technique employs protein chip separates protein mixtures by different surface binding affinity and molecular weights, and roughly identifies proteins through SELDI-TOF MS	ICAT separates proteins by chemical labeling and relative abundance, and then obtains protein identification through ESI MS/MS	IMAC is a powerful protein fractionation method used to enrich metal-associated proteins and peptides, proteins and peptides can be determined by ESI MS/MS

Remarks	Suitable for whole proteome or specific pre-fractioned proteomes, detect large quantity of proteins in a single run, not suitable for low abundant proteins, affected by posttranslational modifications	Simple preparation procedures, sensitive detection limit, small sample requirement, significant results, wide detection range in molecular weight, modified by different surface affinities	Biotinylated tags labeling before analysis; suitable for low abundant proteins; not suitable for post translational modified proteins; more automated	Enrich metal-associated proteins and peptides, easy regeneration, longevity and stability to proteolytic degradation, have to be facilitated by other separation methods, suitable for posttranslational modification

Potential applications	Study drug-induced cellular signaling pathway in a global scale [35, 69]	Identification of drug targets [70]		
	Identification of drug targets [70]	Screen drug candidates [72, 73]	Receptome profiling [75, 76]	Mapping of phosphoproteomes [77, 78] and metalloproteomes [79, 80]
	Study of drug toxicity and side effects [71]	Study of protein-drug interactions [74]		
